# Supplementary figures and images for: MicroRNA-488-3p-loaded engineered exosomes inhibit proliferation, migration and invasion of hepatocellular carcinoma by targeting SEC61G
Source: PLoS One. 2026 Feb 9;21(2):e0341056. doi: 10.1371/journal.pone.0341056 (PMC12885305; doi:10.1371/journal.pone.0341056)

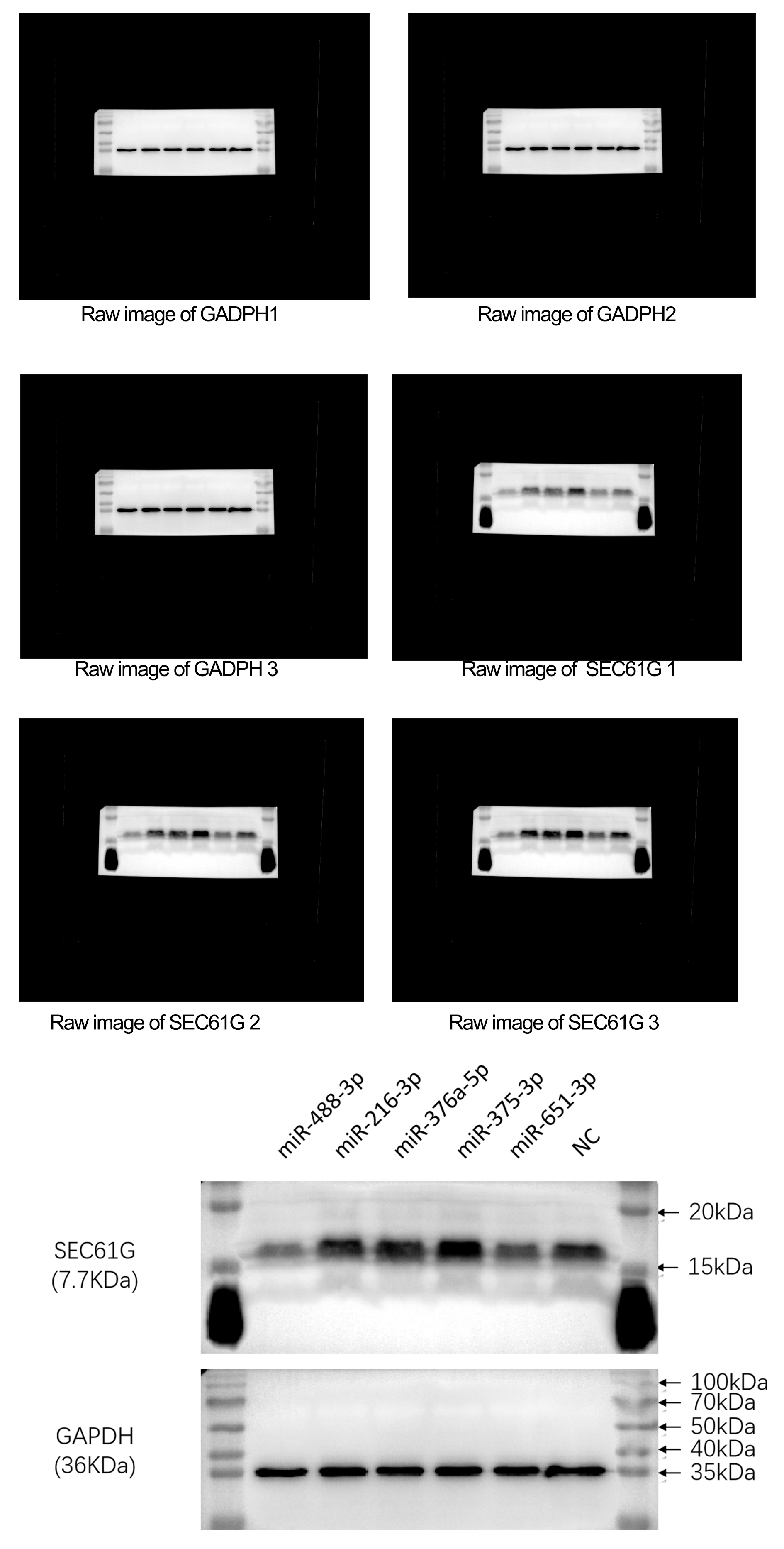

Supplement: S1 Fig — (TIF) [file pone.0341056.s001.tif]

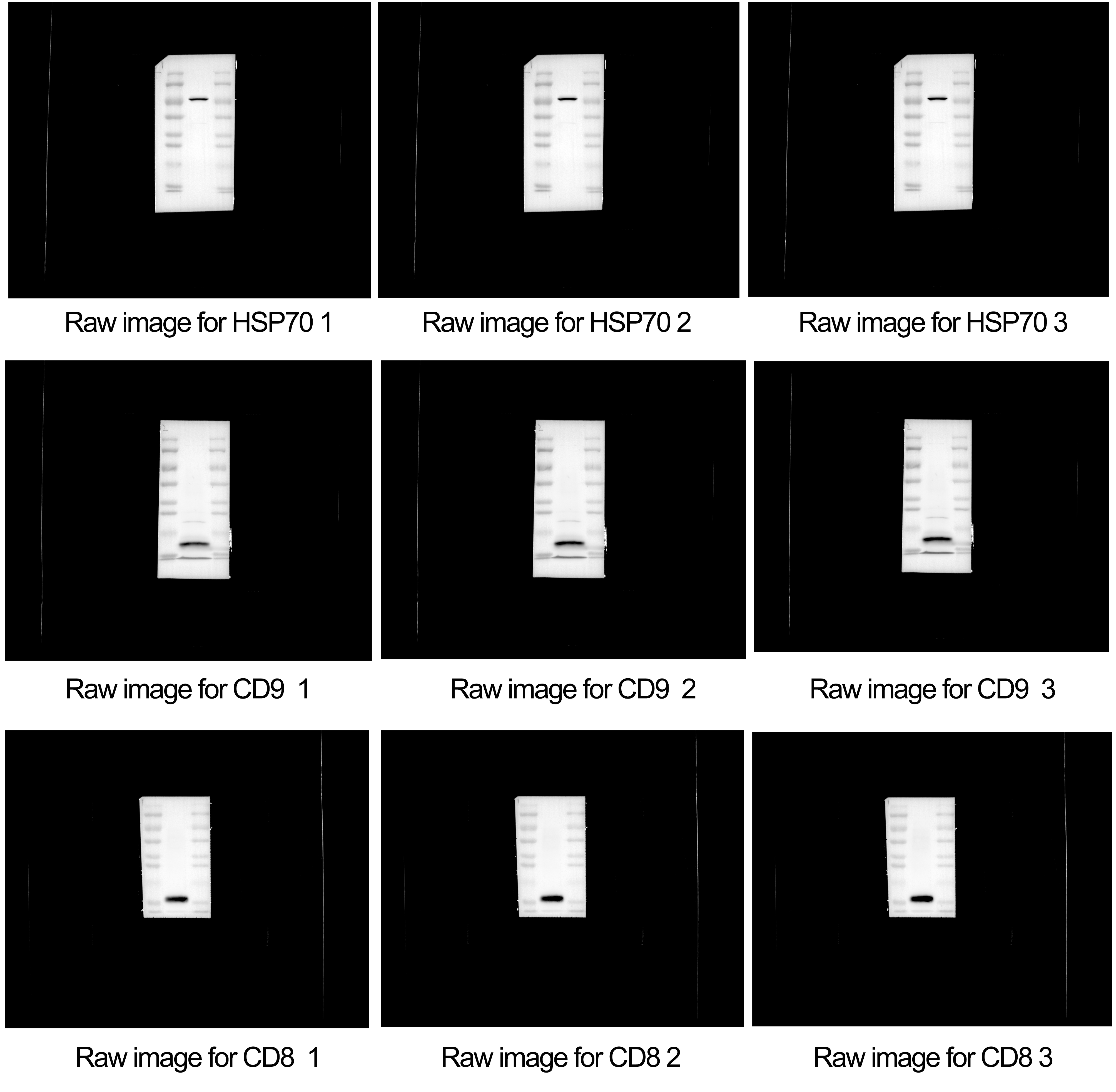

Supplement: S2 Fig — (TIF) [file pone.0341056.s002.tif]
