# Supplementary material for: MicroRNA-488-3p-loaded engineered exosomes inhibit proliferation, migration and invasion of hepatocellular carcinoma by targeting SEC61G
Source: PLoS One. 2026 Feb 9;21(2):e0341056. doi: 10.1371/journal.pone.0341056 (PMC12885305; doi:10.1371/journal.pone.0341056)
Supplement: Attachment — Submitted filename: Response to reviewers.docx [file pone.0341056.s005.docx]

Dear editor,

Thanks for your kind letter about our manuscript (PONE-D-25-40839) entitled “**MicroRNA-488-3p-loaded engineered exosomes inhibit proliferation, migration and invasion of hepatocellular carcinoma by targeting SEC61G**”. We wish to extend our sincere gratitude to you and the reviewers for the time and effort spent on reviewing our manuscript and for providing valuable feedback. We are greatly encouraged by the positive and constructive comments.

We are delighted that the reviewers found the manuscript to be technically sound, statistically rigorous, well-presented, and that the data availability meets the journal's standards.

We have carefully considered all the comments and suggestions provided by the reviewers. In the point-by-point response below, we have addressed each comment in detail and have incorporated all suggested changes into the revised manuscript. The revisions are highlighted in the revised manuscript using the “Track Changes” feature.

We believe that addressing these comments has significantly strengthened the manuscript, and we hope that the revised version now meets the approval of the editorial board and the reviewers.

Sincerely,

Cheng Peng

On behalf of all co-authors

**Journal Requirements:**

**Comment** 1. Please ensure that your manuscript meets PLOS ONE's style requirements, including those for file naming. The PLOS ONE style templates can be found at

https://journals.plos.org/plosone/s/file?id=wjVg/PLOSOne_formatting_sample_main_body.pdf and <https://journals.plos.org/plosone/s/file?id=ba62/PLOSOne_formatting_sample_title_authors_affiliations.pdf>

**Response**: We thank the editorial office for pointing out the journal's style requirements. We have carefully reviewed and confirmed that our revised manuscript now fully complies with PLOS ONE's style policies.

**Comment** 2. Thank you for stating the following financial disclosure:

[This research was funded by the National Natural Science Foundation of China, grant number 82072674; and the Natural Science Foundation of Shandong Province, grant number ZR2020MH258.].

Please state what role the funders took in the study.  If the funders had no role, please state: "The funders had no role in study design, data collection and analysis, decision to publish, or preparation of the manuscript."

If this statement is not correct you must amend it as needed.

Please include this amended Role of Funder statement in your cover letter; we will change the online submission form on your behalf.

**Response**: Thank you for this instruction. As requested, we confirm that the funders had no role in the study design, data collection and analysis, decision to publish, or preparation of the manuscript. The amended "Role of Funder" statement has been added to the ‘Funding Statement’ section in the revised manuscript, as follows:

**Funding**

This work was supported by the National Natural Science Foundation of China, grant number 82072674, and the Natural Science Foundation of Shandong Province, grant number ZR2020MH258. The funders had no role in study design, data collection and analysis, decision to publish, or preparation of the manuscript.

**Comment** 3. Thank you for stating the following in the Acknowledgments Section of your manuscript:

[This research was funded by the National Natural Science Foundation of China, grant number 82072674; and the Natural Science Foundation of Shandong Province, grant number ZR2020MH258.]

We note that you have provided funding information that is currently declared in your Funding Statement. However, funding information should not appear in the Acknowledgments section or other areas of your manuscript. We will only publish funding information present in the Funding Statement section of the online submission form.

Please remove any funding-related text from the manuscript and let us know how you would like to update your Funding Statement. Currently, your Funding Statement reads as follows:

[This research was funded by the National Natural Science Foundation of China, grant number 82072674; and the Natural Science Foundation of Shandong Province, grant number ZR2020MH258.]

Please include your amended statements within your cover letter; we will change the online submission form on your behalf.

**Response**: We thank the editor for this clarification. We have now removed the funding information from the **Acknowledgments section** of the manuscript as requested. The funding details are now only present in the **Funding Statement** section.

The updated Funding Statement for the manuscript is as follows:

**"This research was funded by the National Natural Science Foundation of China, grant number 82072674; and the Natural Science Foundation of Shandong Province, grant number ZR2020MH258. The funders had no role in study design, data collection and analysis, decision to publish, or preparation of the manuscript."**

Please use this amended statement for the online submission form.

**Comment** 4. We note that your Data Availability Statement is currently as follows: [All relevant data are within the manuscript and its Supporting Information files.]

Please confirm at this time whether or not your submission contains all raw data required to replicate the results of your study. Authors must share the “minimal data set” for their submission. PLOS defines the minimal data set to consist of the data required to replicate all study findings reported in the article, as well as related metadata and methods (https://journals.plos.org/plosone/s/data-availability#loc-minimal-data-set-definition).

For example, authors should submit the following data:

- The values behind the means, standard deviations and other measures reported;

- The values used to build graphs;

- The points extracted from images for analysis.

Authors do not need to submit their entire data set if only a portion of the data was used in the reported study.

If your submission does not contain these data, please either upload them as Supporting Information files or deposit them to a stable, public repository and provide us with the relevant URLs, DOIs, or accession numbers. For a list of recommended repositories, please see https://journals.plos.org/plosone/s/recommended-repositories.

If there are ethical or legal restrictions on sharing a de-identified data set, please explain them in detail (e.g., data contain potentially sensitive information, data are owned by a third-party organization, etc.) and who has imposed them (e.g., an ethics committee). Please also provide contact information for a data access committee, ethics committee, or other institutional body to which data requests may be sent. If data are owned by a third party, please indicate how others may request data access.

**Response**: We thank the editor for this important reminder. In accordance with PLOS ONE's data policy, we have now uploaded the complete "minimal data set" as new Supporting Information files.

The uploaded data include:

S1 Fig. Raw western blot images for SEC61G expression after transfected with miRNAs mimics.

S2 Fig. Raw western blot images for exosome markers of CD9, CD81 and HSP70

**Comment** 5. PLOS ONE now requires that authors provide the original uncropped and unadjusted images underlying all blot or gel results reported in a submission’s figures or Supporting Information files. This policy and the journal’s other requirements for blot/gel reporting and figure preparation are described in detail at https://journals.plos.org/plosone/s/figures#loc-blot-and-gel-reporting-requirements and https://journals.plos.org/plosone/s/figures#loc-preparing-figures-from-image-files. When you submit your revised manuscript, please ensure that your figures adhere fully to these guidelines and provide the original underlying images for all blot or gel data reported in your submission. See the following link for instructions on providing the original image data: https://journals.plos.org/plosone/s/figures#loc-original-images-for-blots-and-gels.

In your cover letter, please note whether your blot/gel image data are in Supporting Information or posted at a public data repository, provide the repository URL if relevant, and provide specific details as to which raw blot/gel images, if any, are not available. Email us at plosone@plos.org if you have any questions.

**Response**: We thank the editor for noting this policy. We have provided the original underlying images for the majority of our blot/gel results as Supporting Information files.

**Comment** 6. Please include your full ethics statement in the ‘Methods’ section of your manuscript file. In your statement, please include the full name of the IRB or ethics committee who approved or waived your study, as well as whether or not you obtained informed written or verbal consent. If consent was waived for your study, please include this information in your statement as well.

If the reviewer comments include a recommendation to cite specific previously published works, please review and evaluate these publications to determine whether they are relevant and should be cited. There is no requirement to cite these works unless the editor has indicated otherwise.

**Response**: Thank you for this instruction. We confirm that our study did **not** involve any of the data of human participants, human tissue and animal experiments. Therefore, our research did not require review or approval by an Institutional Review Board (IRB) or ethics committee.

**Additional Editor Comments**:

Thank you for your patience and for considering our journal for your interesting manuscript. We have now received the reviewers’ comments, which are included below. Based on their feedback, we kindly request that you revise the manuscript by addressing these points carefully. Please do not hesitate to reach out if you have any questions or need clarification.

**Reviewers' comments:**

**Comments to the Author**

**Comment** 1. Is the manuscript technically sound, and do the data support the conclusions?

The manuscript must describe a technically sound piece of scientific research with data that supports the conclusions. Experiments must have been conducted rigorously, with appropriate controls, replication, and sample sizes. The conclusions must be drawn appropriately based on the data presented.

Reviewer #1: Yes

Reviewer #2: Yes

**Response**: We sincerely thank the reviewers for their positive assessment and for confirming that the manuscript is technically sound and the conclusions are well-supported by the data.

**Comment** 2. Has the statistical analysis been performed appropriately and rigorously?

Reviewer #1: Yes

Reviewer #2: Yes

**Response**: We thank the reviewers for their positive feedback and for confirming the appropriateness and rigor of the statistical analysis.

**Comment** 3. Have the authors made all data underlying the findings in their manuscript fully available?

The PLOS Data policy requires authors to make all data underlying the findings described in their manuscript fully available without restriction, with rare exception (please refer to the Data Availability Statement in the manuscript PDF file). The data should be provided as part of the manuscript or its supporting information, or deposited to a public repository. For example, in addition to summary statistics, the data points behind means, medians and variance measures should be available. If there are restrictions on publicly sharing data—e.g. participant privacy or use of data from a third party—those must be specified.

Reviewer #1: Yes

Reviewer #2: Yes

**Response**: We are pleased that the reviewers have confirmed our compliance with PLOS ONE's data availability policy, and we thank them for their validation.

**Comment** 4. Is the manuscript presented in an intelligible fashion and written in standard English?

PLOS ONE does not copyedit accepted manuscripts, so the language in submitted articles must be clear, correct, and unambiguous. Any typographical or grammatical errors should be corrected at revision, so please note any specific errors here.

Reviewer #1: Yes

Reviewer #2: Yes

**Response**: We thank the reviewers for their positive assessment of the manuscript's clarity and standard English presentation.

**Comment** 5. Review Comments to the Author

Please use the space provided to explain your answers to the questions above. You may also include additional comments for the author, including concerns about dual publication, research ethics, or publication ethics. (Please upload your review as an attachment if it exceeds 20,000 characters).

**Reviewer #1**:

1) Line 117 The medium name is written as "DEME". Should this be "DMEM"?

**Response**: We thank the reviewer for the careful reading and for identifying this typographical error. We have now corrected "DEME" to "DMEM" (Dulbecco's Modified Eagle Medium) in the revised manuscript on line 118.

2) Line 128

It might be helpful to include a description of the quantification method used for the isolated exosomes.

**Response**: We thank the reviewer for this valuable suggestion. As suggested, we have now added a description of the exosome quantification method in the revised manuscript on lines 129-130. The added text reads: “The particle concentration and size were determined using nanoparticle tracking analysis (NanoSight NS300, Malvern Panalytical). The exosomes were then stored at -80 °C for use.”

3) Line 171 The product name appears to be outdated. Should this be updated to "ECL Prime"? If the name differs depending on the sales region, forget about it.

**Response**: We thank the reviewer for pointing this out. We have verified the product used and revised the name as “ECL-plusTM kit” in the manuscript. In addition, we have added the manufacturer's information (e.g., Amersham, Piscataway, NJ, USA) to the Methods section for greater clarity. We are grateful for the reviewer's meticulous comment.

4) Line 247

Just to confirm: Is my understanding correct that miR-488-3p was only effective when loaded into exosomes?

**Response**: Thanks for reviewer’s constructive suggestion. Our results determined that the transfection of miR-488-3p mimics by Lipofectamine 2000 significantly reduced the cell viability of HepG2 cells and the expression of SEC61G. Exosomes, as a class of extracellular vesicles, play important roles in cell-cell communications ^[9]^ and have emerged as promising endogenous nanomaterials/carriers for drug delivery and therapy ^[13]^. Given the growing interest in the clinical application of exosomes in HCC treatment, we specially focused on evaluating the therapeutic potential of exosome-delivered miR-488-3p. Our data conclusively showed that exosomal miR-488-3p effectively suppressed HepG2 cell proliferation, migration and invasion. While we recognize that a direct comparison with free miRNA could offer further insights, the core and novel contribution of our study lies in establishing the efficacy of an exosome-based delivery system for miR-488-3p.

5) Line 291

For consistency, would it be possible to label the images in Figure 4B and 4E with "Control", "Exo-NC", and "Exo-miR-488-3p", similar to Figure 5?

**Response**: We thank the reviewer for this excellent suggestion. As suggested, we have now updated the labels in Figure 4B and 4E to "Control", "Exo-NC", and "Exo-miR-488-3p" to align with the labeling convention used in Figure 5. The revised figures are listed below:

**Reviewer #2**:

1) Line 107 and 109- Please can the authors capitalize the whole primer sequence and maintain consistency throughout the manuscript.

**Response**: We thank the reviewer for this suggestion. We have now capitalized the entire sequence of all primers (both the ones mentioned here and others throughout the manuscript) to maintain consistency. The changes are shown below:

LV-pCDH-miR-488-3p-Xba/Not-F: 5’- ACCTCCATAGAAGATTCTAGAGGCTCTATGGAACAAAACCAGTTT-3’, LV-pCDH-miR-488-3p-Xba/Not-R 5’- GATCGCAGATCCTTCGCGGCCGCAGGGGTACATATAGTATCCATCTTTTCA-3’.

2) Line 80- Mention the full form of DMEM and then abbreviate in the manuscript

**Response**: We thank the reviewer for their valuable comments. We have now defined the abbreviation by spelling out the full term Dulbecco's Modified Eagle Medium (DMEM) at its first occurrence in the manuscript, as per the comment. The abbreviation is used thereafter.

3) Line 117- DEME is mentioned instead of DMEM

**Response**: We thank the reviewer for pointing out this typographical error. We have revised "DEME" to "DMEM" in Line 118 of the revised manuscript.

4) Figure 4- Please can the authors label the diagrams B and E with study groups.

**Response**: We thank the reviewer for the suggestion. The diagrams in Figure 4B and 4E have been labeled with the study groups as requested. The changes are listed as below:

5)Please can authors provide an explanation why miR-448-3p activity and effectiveness was not looked at throughout the study?

**Response**: Thanks for reviewer’s constructive suggestion. During our careful re-examination of the data in response to the reviewer's comments, we identified and corrected an error in the data compilation for the apoptosis assay. The initial analysis inadvertently showed no significant difference between the Exo-NC and Exo-miR-488-3p groups. The initial analysis inadvertently showed no significant difference between the Exo-NC and Exo-miR-488-3p groups. We sincerely apologize for this oversight. We have now re-analyzed the data correctly. The updated results conclusively demonstrate that Exo-miR-488-3p significantly promotes apoptosis in HepG2 cells compared to the Exo-NC group, which is consistent with its inhibitory effects on proliferation, migration, and invasion. Figure 4D and E, and the corresponding text in the Results section have been updated accordingly in the revised manuscript. The revised Figure 4 is listed as follows:

6) Please can the authors mention how many times the experiments were performed in the figure legend or results.

**Response**: We thank the reviewer for this valuable suggestion to improve the clarity and rigor of our manuscript. As recommended, we have now explicitly stated the number of independent experimental replicates (n=3) for all quantitative data in the figure legends of Figures 1, 4, and 5. The legends for **Figure 1, 4, and 5** have been revised to include the statement: "Data are presented as the mean ± SD from three independent experiments".

7) Figure 1- Is it possible for the western blot to be repeated, the miR-375-3p has two bands, please can you provide an explanation.

**Response**: Thanks for reviewer’s kind suggestion. We agree that the presence of a non-specific band could cause ambiguity. In response, we have carefully repeated the western blot experiment for all miRNA mimics, including miR-375-3p, under optimized and stringent conditions. The new, reproducible results clearly show that transfection with the **miR-375-3p mimic yields a single, specific band for SEC61G**, which aligns with the expected molecular weight. The original appearance of two bands was likely due to a non-specific antibody reaction or minor experimental variability in that particular run. We have revised Figure 1B, as below:

8) Figure 4 A- cell viability just has two lines for 3 groups, can you change it to color or make the third group prominent.

**Response**: We thank the reviewer for the careful review. As recommended, we have colored the lines of control and Exo-NC group to make the third group prominent. The revised Figure is listed below:

9) Figure 4C- there is no conclusive evidence that the apoptosis numbers were elevated since there is no statistical significance, the authors can confirm this by performing western blot for apoptotic markers like Bax, BCl2, PARP.

**Response**: Thanks for reviewer’s constructive suggestion. During our careful re-examination of the data in response to the reviewer's comments, we identified and corrected an error in the data compilation for the apoptosis assay. We sincerely apologize for this oversight. We have now re-analyzed the data correctly. The updated results conclusively demonstrate that Exo-miR-488-3p significantly promotes apoptosis in HepG2 cells compared to the Exo-NC group, which is consistent with its inhibitory effects on proliferation, migration, and invasion. Figure 4D and E, and the corresponding text in the Results section have been updated accordingly in the revised manuscript. The revised Figure 4 is listed as follows:

10) Figure 5- scale bars are missing in the image. Also, some graphs are bordered and some are not, please try to maintain consistency throughout the figures.

**Response**: Thanks for the reviewer’s careful review. We have added clear scale bars to all representative images in Figure 5 and unified the formatting of all graphs in Figure 5 to ensure consistency. The revised figure is listed below:

6. PLOS authors have the option to publish the peer review history of their article (what does this mean?). If published, this will include your full peer review and any attached files.

If you choose “no”, your identity will remain anonymous but your review may still be made public.

Do you want your identity to be public for this peer review? For information about this choice, including consent withdrawal, please see our Privacy Policy.

Reviewer #1: No

Reviewer #2: No

**Response**:

No
