# Supplementary material for: MicroRNA-488-3p-loaded engineered exosomes inhibit proliferation, migration and invasion of hepatocellular carcinoma by targeting SEC61G
Source: PLoS One. 2026 Feb 9;21(2):e0341056. doi: 10.1371/journal.pone.0341056 (PMC12885305; doi:10.1371/journal.pone.0341056)
Supplement: Attachment — Submitted filename: response to reviewer.doc [file pone.0341056.s006.doc]

Dear Dr. Anisha D’Souza,

Thank you for handling our manuscript (**PONE-D-25-40839R1**) entitled “**MicroRNA-488-3p-loaded engineered exosomes inhibit proliferation, migration and invasion of hepatocellular carcinoma by targeting SEC61G**” and for your positive feedback. We are pleased that the reviewers found our previous revisions satisfactory and that only a minor typographical correction is required. We have carefully addressed the comment below and have prepared the revised manuscript accordingly.

We believe that the manuscript now fully complies with PLOS ONE’s formatting and editorial standards. Thank you once again for your valuable time and guidance throughout the review process.

Sincerely,

Cheng Peng

**Reviewer #1**:
Comment: line 175: Please ensure that the trademark symbol in "ECL-plus™ kit" is formatted as a superscript (™) according to standard typographic conventions. This is a minor formatting issue but should be corrected for consistency.
**Response**: Thank you for this careful observation. We have corrected the trademark symbol “™” to superscript format in line 175 of the revised manuscript, as suggested.

**Reviewer #2**:
**Comment**: All comments have been addressed.
**Response**: We thank the reviewer for confirming that all previous comments have been adequately addressed.
